# Is the Presence of the Father of the Baby during First Prenatal Ultrasound Study Visit Associated with Improved Pregnancy Outcomes in Adolescents and Young Adults?

**DOI:** 10.1155/2016/4632628

**Published:** 2016-10-04

**Authors:** Sara H. Lee, Rina Lazebnik, Margaret Kuper-Sassé, Noam Lazebnik

**Affiliations:** ^1^Department of Pediatrics, Case Western Reserve University School of Medicine, Cleveland, OH, USA; ^2^Department of Obstetrics and Gynecology, Case Western Reserve University School of Medicine, Cleveland, OH, USA

## Abstract

This study examined whether the presence of the father of the baby (FOB) at the first prenatal ultrasound study (US) visit of pregnant adolescents and young adults (AYA) is a marker for improved pregnancy outcomes. Charts of 400 pregnant AYA aged 14–22 years seen at an academic maternity hospital were assessed retrospectively for support persons brought to prenatal US visits. Logistic regression analysis was used to examine the association between FOB presence and gestational age and birth weight. Of 400 charts with support person recorded, 298 charts with first US visit data, singleton birth, and complete gestational data available were analyzed. FOB was present at 30.2% of visits, while the parent of the mother was present at 34.2% of visits. With FOB present, 3.3% of infants were born preterm (gestational age < 37 weeks) compared with 10.5% of infants with FOB absent (*p* = 0.04). Patients with FOB present also had significantly earlier gestational age at the first US visit (15 weeks) than those who did not (19 weeks; *p* = 0.02). For AYA, the presence of FOB at initial prenatal US visits is a predictor of improved pregnancy outcome and likely represents increased support during the pregnancy.

## 1. Introduction

Even though the national teen pregnancy rate is declining [1], adolescent pregnancies continue to be a problem for individuals and society [2]. Adolescents are at risk for a number of pregnancy complications, including preterm labor, premature delivery, low birth weight infants, and maternal morbidity [3], all of which can contribute to both immediate and long-term costs. Also, teens who have had a child are more likely to drop out of high school or be incarcerated, and the children of teen mothers also have lower educational attainment and higher incarceration rates [4, 5].

Inadequate prenatal care for adolescents is strongly associated with prematurity [6], and adolescents are more likely than older women to receive late or no prenatal care [7]. Explanations for this discrepancy include difficulty accessing health care, unplanned or denied pregnancy, and lack of social supports [8]. Feldman found that support expectation, “knowing that others would be available after the birth of the child,” predicted prenatal attachment between the adolescent mother and infant during pregnancy and hypothesized that improved prenatal attachment would lead to increased prenatal care [9]. Social support interventions have been shown to reduce the incidence of low birth weight in adult at-risk populations [10].

Shah et al. recently looked at the effect of partner support on birth outcomes in pregnant adolescents and, using a secondary analysis of survey data, found that teens with a supportive or involved partner were less likely to have adverse outcomes such as low birth weight or pregnancy loss [11]. Few studies have looked at the presence of a support person during the adolescent's pregnancy. A survey of genetic counselors noted that teens were more likely to be accompanied by a parent, friend, or sibling as compared to adult women who were usually alone, with the father of the pregnancy, or with a significant other person [12].

Ultrasound study visits are a visual confirmation of pregnancy. An ultrasound study also offers the opportunity for a support person to be with the pregnant teen. We hypothesized that the presence of the father of the baby at the first ultrasound visit would be associated with improved pregnancy outcomes, specifically birth weight and pregnancy duration. While first ultrasounds are recommended at our center to be performed at 12-13 weeks, the tendency of AYA to have later diagnosis of pregnancy and later prenatal care contributed to the choice of first ultrasound visit for this study as some women would not have a subsequent ultrasound. Extensive literature search failed to find any study aimed at finding possible correlations between the presence of the father of the baby at adolescent prenatal ultrasound study visits and pregnancy outcomes.

## 2. Methods

Approval was obtained from the Institutional Review Board. A retrospective chart review was conducted at an academic maternity and women's hospital located in a Midwestern city in the United States that serves women who receive both private insurance and Medicaid. The hospital has approximately 4,500 deliveries per year. The charts of pregnant adolescents and young adults seen over a two-year and a year and half period were assessed. Inclusion criteria were support person documented (presence or absence) and age between 12 and 22 years at first ultrasound study (US) visit. Twin pregnancies were excluded.

Data collected included

- support person and relationship type;
- gestational age at visit;
- pregnancy complications;
- gestational age at delivery;
- birth weight at delivery;
- birth complications.

Additional demographic data was not available.

Logistic regression analysis was used to examine the association between the presence of support persons at prenatal US visits and gestational age. Statistical analysis was conducted using SAS 9.3.

## 3. Results

Over 1400 charts were reviewed (*n* = 1444); 400 had the presence or absence of support person(s) noted. Support person is not a required field in patient charts. A total of five charts were excluded because the first US visit was not available, and additional 6 charts were excluded because of twin pregnancies. A total of 298 charts had gestational age at delivery recorded, and 205 charts had birth weight recorded.

Maternal and pregnancy characteristics are detailed in Table 1.

The mean maternal age was 18.3 years with a range of 14 to 22 years. Mean gestational age by ultrasound at first visit was 18.1 years with a range of 5 to 40 weeks. Mean gestational age at birth was 38.6 weeks with a range of 23 to 42 weeks. Mean birth weight was 3800 grams with a range of 440 to 4600 grams. Preterm birth occurred in 8.4% of deliveries, and 9.8% of infants were with low birth weight.

**Figure 1:**
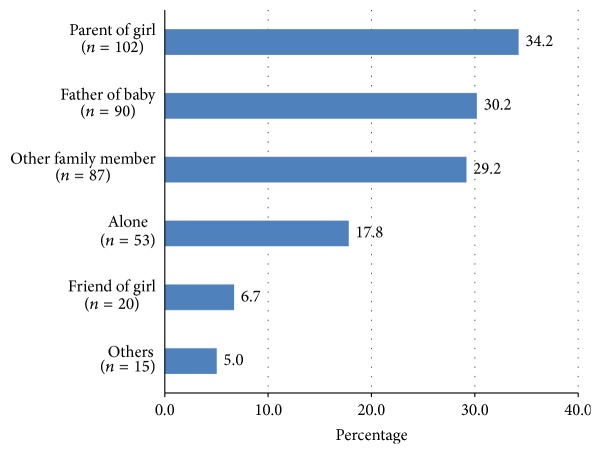
Support person present at first ultrasound visit.

The majority of adolescents and young adults were accompanied by a parent (34.2%) or the father of the baby (30.2%). Other support persons included family members and friends, and 17.8% of pregnant adolescents and young adults were alone in the visit (Figure 1). The majority of pregnant adolescents and young adults came accompanied by one person (61.1%); however, 21.1% came with two or more people to the US visit.

**Figure 2:**
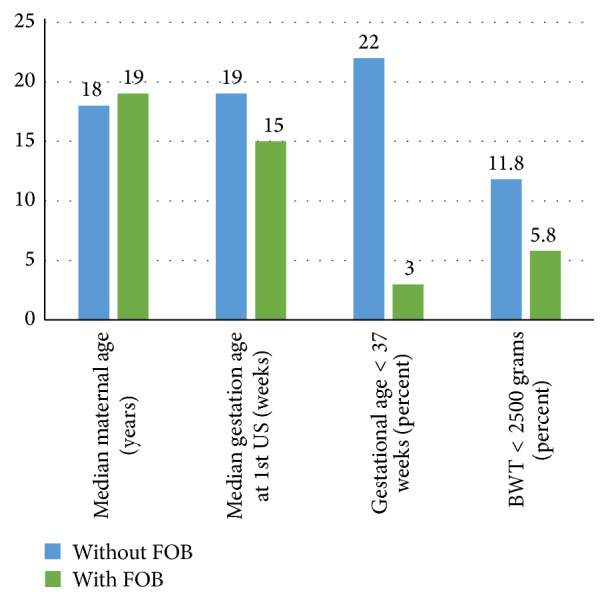
Association of the presence of father of baby with prenatal and pregnancy outcomes.

There was a significant association between the presence of the father of the baby at the first US visit and full term birth. With the father of the baby present, 3.3% of infants were born preterm (gestational age < 37 weeks) compared with 10.5% of infants with the father of the baby absent (*p* = 0.04). Adolescents and young adults with the father of the baby present also presented for first US at a significantly earlier gestational age (15 weeks versus 19 weeks, *p* = 0.02). Fewer infants with the father of the baby present at first US were with low birth weight, but this was not a significant difference (5.8% versus 11.8%, *p* = 0.22) (Figure 2).

## 4. Discussion

To our knowledge this study is one of the first to look at the association between the father of the baby at adolescent and young adult ultrasound study visits and pregnancy outcomes. Multiple studies have looked at paternal support (and support of family and peers) in adolescent and adult pregnant women; however, few have looked at the actual presence of these support people at any prenatal visit [13]. One of the strengths of this study lies in the use of documented support person presence or absence rather than other proxies of support.

This study found that the presence of the father of the baby at the adolescent and young adult prenatal US visit was associated with lower rates of preterm delivery. Studies in adult women have found that paternal support may decrease stress in pregnant women which in turn may decrease poor birth outcomes [14]. Stress and anxiety are both risk factors for preterm birth and low birth weight [15]. The presence of the father of the baby may also indicate a desired pregnancy, which may in itself be less stressful and indicate future partnership and involvement. Partner support for adult pregnant women has also been shown to encourage healthier maternal behaviors, such as smoking cessation and decreased alcohol consumption [16]. Those behaviors were not assessed in our study; however, decreased risky behaviors could have contributed to the positive outcomes.

We also found that the presence of the father of the baby at the adolescent and young adult prenatal US visit was associated with earlier gestational age at the time of the first ultrasound. An early US implies that prenatal care has begun early in the pregnancy. Improved prenatal care may help to explain the improved birth outcomes. The involvement of the father of the baby might lead to earlier acceptance and acknowledgement of the pregnancy. Also, the father of the baby may be able to help in the decision-making that occurs at US visits. Pregnant adolescents and young adults are more likely to make noninformed decisions about prenatal screening [17]. However, fathers who attend ultrasounds report feeling that they react to information objectively and are able to make decisions [18]. Paternal presence may help with adherence to recommended prenatal testing.

This study does have several limitations including an absence of outcome data due to changing documentation system. Also, many charts were excluded because the presence or absence of a support person was not recorded, leading to an overall small number of charts included.

Future directions would include both quantitative and qualitative studies to examine the role of fathers in pregnancy outcomes. Interviews with adolescents and young adult parents would help give insight into paternal engagement and its potential influence on maternal and infant outcomes.

In conclusion, the presence of the father of the baby at the prenatal US visits of adolescents and young adults is associated with improved pregnancy outcomes. The presence of father of the baby at adolescent and young adult prenatal US visits is also associated with earlier gestation at first ultrasound and may imply better prenatal care. Interventions to encourage paternal involvement may lead to improved pregnancy outcomes for adolescents and young adults.

## Figures and Tables

**Table 1:** Adolescent and young adult ages and pregnancy characteristics.

	Mean	Standard deviation	Range
Maternal age (years)	18.3	2.1	14–22
Gestational age at visit (weeks)	18.1	7.3	5–40
Gestational age at birth (weeks)	38.6	2.3	23–42
Birth weight (grams)	3080	577	440–4600
